# Analysis of Clinicopathological Characteristics and Its Correlation With the Prognosis of Pediatric Lupus Nephritis: A Tertiary Care Center Experience

**DOI:** 10.7759/cureus.21862

**Published:** 2022-02-03

**Authors:** Barathi G, Mahesh Janarthanan, Indhuumathy Thayammal S, Subalakshmi Balasubramanian, Sangeetha Geminiganesan

**Affiliations:** 1 Department of Pathology, Sri Ramachandra Institute of Higher Education and Research, Chennai, IND; 2 Department of Rheumatology and Division of Pediatric Rheumatology, Sri Ramachandra Institute of Higher Education and Research, Chennai, IND; 3 Department of Paediatric Medicine, Sri Ramachandra Institute of Higher Education and Research, Chennai, IND; 4 Department of Pediatric Nephrology, Sri Ramachandra Institute of Higher Education and Research, Chennai, IND

**Keywords:** chronic kidney disease, chronicity index, activity index, lupus nephritis, systemic lupus erythematosus

## Abstract

Aim

To study the various pathological patterns of pediatric lupus nephritis (LN) by renal biopsies and to correlate the histopathological data with the clinical and biochemical outcomes.

Methods

This is a retrospective study in children between 1 month and 18 years of age with renal biopsy-proven lupus nephritis, conducted between January 2015 and December 2019. Various pathological and clinical parameters were compared between the groups with lupus nephritis activity and those without activity.

Results

Of 38 biopsy-proven lupus nephritis cases, 30 (78.9%) were in the adolescent age group, and the female gender was predominantly affected (n=30; 78.9%). Class IV proliferative lupus nephritis (n=17, 44.7%) was the most common biopsy finding, and the activity score for endocapillary hypercellularity, neutrophil infiltration, fibrinoid necrosis, hyaline deposits, and interstitial inflammation was significantly high in classes III and IV. Overall, attaining remission was less, and the risk of progression of chronic kidney disease (CKD) was higher in class IV (n=3; 7.8%). Mortality was reported in 1 out of 38 (2.6%) children.

Conclusion

Light microscopy and immunofluorescence studies play an important role in defining the extent of renal damage in the form of activity and chronicity indices, which are the key factors in the decision-making of lupus nephritis treatment. The prognostic relevance of the histological scoring has been evaluated, and it is evident that the activity index and chronicity index go a long way in therapeutic intervention.

## Introduction

Systemic lupus erythematosus (SLE) is a chronic multisystem autoimmune disease. The crude incidence rate ranges from 0.9 to 3.1 (per 100,000 per year) across Asia-Pacific countries, while the prevalence rate ranges between 4.3 and 45.3 (per 100,000 per year) [1]. In SLE, patients who develop lupus nephritis (LN) present at a younger age than patients without nephritis [2]. In pediatric patients with SLE, one of the most common organs involved is the kidneys. Previous studies have shown that up to 80% of children with SLE eventually develop nephritis. They may either present with overt renal symptoms at the time of diagnosis (21-65%) or during the course of their illness (40-82%) [1].

The advent of newer drugs and regimens has paved the way for the early initiation of treatment following the diagnosis of LN, leading to a more chronic course of the disease with the maintenance of renal function in adults. Compared to their adult counterparts, children with LN have a more active disease course and poorer outcomes, with an increased rate of progression to end-stage renal disease (ESRD), compromising the quality of life in many children [3]. Renal involvement in SLE is the major determinant of long-term survival and morbidity in children. There is a need for a good prognostic indicator. Only a few studies are available on the prognosis of pediatric LN in India, with most being from the northern and eastern parts of the country [4-6]. There is a paucity of data on this subject from south India. This study has been designed to determine the correlation between the pathological, biochemical, clinical, and treatment outcomes of children diagnosed with lupus nephritis in a tertiary care center located in the southern part of India.

## Materials and methods

Aims and objectives of the study

The objectives of this study are to study the demographic data, clinical features, biochemical parameters, and other serological values in paediatric lupus nephritis; to evaluate the various pathological patterns of paediatric lupus nephritis in renal biopsies and analyze the significance of activity and chronicity indices with various demographic parameters; and to correlate histopathological data with clinical and biochemical outcome in follow-up patients.

Materials and methodology

It is a retrospective, cross-sectional, single-centre study conducted after obtaining the Institutional Ethics Committee approval (IEC-NI/21/FEB/77/30) in the Department of Paediatric Nephrology with collaborative efforts from the Department of Pathology and the Department of Paediatric Rheumatology in a tertiary care centre in South India. Of 110 children diagnosed with SLE between January 2015 and December 2019 in our institute, we retrieved the follow-up data from 38 children with biopsy-proven lupus nephritis.

Inclusion criteria

Children under 18 years of age with lupus nephritis were included in the study. In our study, the updated EULAR/ACR (European League Against Rheumatism/American College of Rheumatology) 2019 revised classification criteria for the diagnosis of SLE were used [7].

Exclusion criteria

We planned to exclude children with transplanted kidneys, children with involvement of the kidneys on the background of any other chronic diseases, and children with inconclusive results of missed renal biopsy from the study. However, there were no such patients in our cohort.

Data collection

Data were obtained from the medical records department, including demography, clinical features, and investigations. At the time of the renal biopsy, spot urine protein creatinine ratio (uPCR), 24-hour urine protein, complete blood count (CBC), renal function test (RFT), complement C3, C4, antinuclear antibody (ANA), serum double-stranded DNA (dsDNA), and anti-phospholipid antibodies were noted. Data pertaining to urine routine, urine protein to creatinine ratio, blood urea nitrogen, and serum creatinine were also collected at 6 months and 12 months post-renal biopsy. Proteinuria was defined as urine protein ≥1+ (30 mg/dL) by dipstick test and spot urine protein creatinine ratio >0.2 mg/mg. More than 500 mg/day of protein in the urine over 24 hours was a prerequisite for renal biopsy. Microscopic hematuria was defined as >5 red blood cells/high power field and active urinary sediments such as red blood cell casts were noted. Complete remission was defined as the resolution of proteinuria with absent active urinary sediments and microscopic hematuria. Chronic kidney disease (CKD) stages were defined with estimated glomerular filtration rate (eGFR). eGFR of 30 to 59, 15 to 29, and less than 15 were noted as stages 3, 4, and 5 CKD, respectively. On follow-up, uPCR of 0.2 to 2 mg/mg was noted as nephritic range proteinuria and uPCR of >2 mg/mg was taken as nephrotic range proteinuria.

Histopathology data

Renal biopsies were done under ultrasound guidance using an 18 gauge, 22 mm × 10 cm semi-automated renal biopsy gun. All renal biopsy specimens were examined by the pathologist using a light microscope and an indirect immunofluorescence microscope. As per the standard operating procedure, hematoxylin and eosin, periodic acid Schiff, Jones Methenamine silver, and Masson’s Trichrome stains were performed for light microscopy. Specimens for immunofluorescence microscopy were stained using fluorescein isothiocyanate (FITC) conjugated polyclonal rabbit antisera against human IgG, IgM, IgA, C3c, C1q, kappa, lambda, fibrinogen, and albumin. Immunofluorescence findings were categorized based on location and intensity, from (+) to (++++). The International Society of Nephrology/Renal Pathology Society (ISN/RPS 2003) criteria with NIH (National Institute of Health) update were applied for scoring the activity and chronicity index in proliferative lupus nephritis classes III and IV [8]. Indicators of disease activity include endocapillary hypercellularity, neutrophils and karyorrhexis within the glomerular capillary loops, fibrinoid necrosis, hyaline deposits, cellular or fibro-cellular crescents, and interstitial inflammation. Indicators of disease chronicity include the total percentage of global glomerulosclerosis, fibrous crescents, tubular atrophy, and interstitial fibrosis. The scoring was given as 0 to 3, based on the percentage of glomeruli involved. Scores of 0 indicated no glomerular involvement, while scores of 1, 2, and 3 indicated 25%, 25-50%, and >50% glomerular involvement, respectively.

Treatment design

The treatment plan for each patient was made based on activity and chronicity index, in line with the NIH guidelines [9]. All children received pulse injections of methylprednisolone or oral prednisolone in the intensive phase. Lupus nephritis classes I and II were treated with either azathioprine or mycophenolate mofetil along with steroid tapering whereas classes III and IV received injection cyclophosphamide monthly pulses for six months. Parents/caretakers of the patients were counselled about the disease, its effect on the kidneys, adverse effects of the medications, and long-term outcomes. Patients were reviewed on a fortnightly/monthly basis or as deemed necessary.

Statistical analysis

Data were analyzed using SPSS software (IBM Corp., Armonk, NY). Results were compared between lupus nephritis with activity (class III, IV, and combined IV+V) and without activity (class I, II, and V). Analysis was expressed as numbers, percentages, and mean/standard deviation (SD) as appropriate and also in terms of the significance of association at a 95% confidence level with a "p-value" of less than 0.05 as significant. A box and whisker plot was used to substantiate the interquartile range for both the activity and chronicity indices in classes III and IV lupus nephritis.

## Results

In our study, 38 biopsy-proven lupus nephritis cases were studied. The study population was divided age-wise into four groups, no patients in the age group of less than 5 years, 5 years to <10 years (n=8; 21%), 10 years to <15 years (n=12; 31.6%), and 15 years to <18 years (n=18; 47.4 %). Female children were affected more (n=30; 78.9%) with the male to female ratio of 2:7.5. Eight children (n=8; 21%) presented with renal manifestations in the form of proteinuria or elevated creatinine during the diagnosis of SLE, whereas 30 (79%) developed consecutively. The clinical presentation has been summarized in Table 1.

**Table 1:** Clinical presentation and laboratory parameters of lupus nephritis

Symptoms and clinical characteristics	Number of patients (%)
Skin – malar rash and oral ulcer Alopecia	10 (26.3%), 5 (13.1%)
Hypertension	10 (26.3%)
Anasarca	12 (31.5%)
Cardiovascular system	2 (5.3%) mitral regurgitation -1, pericardial effusion - 1
Central nervous system	2 (5.3%) hypertensive seizures - 1, intractable headache - 1
Hematology	2 (5.3%) deep vein thrombosis - 1, pulmonary thromboembolism - 1
Abdomen (pancreatitis)	2 (5.3%)
Sepsis	1 (2.6%)
ANA	38 (100%)
Ds DNA	38 (100%)
Complements (C3, C4)	Low in 38 (100%)
APLA	3 (7.9%)
Proteinuria	38 (100%)
Hematuria	7 (17.07%)
Elevated creatinine	3 (7.9%)

Hypocomplementemia was seen in all patients, and the mean C3 and C4 were 47.19 and 8.36, respectively. ANA and anti-dsDNA were positive in all patients (n=38, 100%). Antiphospholipid antibodies (APLA) were positive in 3 (7.9%).

Lupus nephritis staging was done using ISN-RPS classification, which showed classes I (n=3; 7.9%), II (n=9; 23.7%), III (n=9; 23.7%), and IV including the combined class V LN (n=17, 44.7%). The majority were in the class IV group (n-14, 36.8%). There was no pure class V lesion in our study group. The patients were further grouped as having lupus nephritis with activity (n-26; 68.4%) and without activity (n-12; 31.6%). Histopathological findings of both groups are given in Table 2.

**Table 2:** Lupus nephritis histopathological findings

Lupus nephritis with activity	Endocapillary proliferation		Neutrophils		Fibrinoid necrosis		Hyaline deposits		Crescents		Interstitial inflammation		Global sclerosis		Tubular atrophy		Interstitial fibrosis		Arterial lesion	
	+	Nil	+	Nil	+	Nil	+	Nil	+	Nil	+	Nil	+	Nil	+	Nil	+	Nil	+	Nil
Class III	7	2	9	0	4	5	6	3	0	9	7	2	3	6	7	2	5	4	2	7
Class IV	11	1	10	2	4	8	5	7	1	11	6	6	2	10	4	8	3	9	3	9
Class IV+ V	5	0	5	0	5	0	4	1	1	4	5	0	4	0	4	1	4	1	3	2
Lupus nephritis without activity																				
Class I	0	3	0	3	0	3	0	3	0	3	0	3	0	3	0	3	0	3	1	2
Class II	0	9	0	9	0	9	0	9	0	9	0	9	0	9	0	9	0	9	2	7

Pathological findings were compared between the lupus nephritis active and non-active groups and are shown in Table 3.

**Table 3:** Comparison of renal biopsy findings between lupus nephritis active and non-active group

Pathological findings	Lupus nephritis with active disease	Lupus nephritis without active disease	p-Value
Endocapillary proliferation	23	0	0.001
Neutrophil infiltration	24	0	0.001
Fibrinoid necrosis	13	0	0.003
Hyaline deposits	15	0	0.001
Crescents	2	0	0.324
Interstitial inflammation	18	0	0.001
Global sclerosis	6	0	0.07
Tubular atrophy	15	0	0.001
Interstitial fibrosis	12	0	0.004

**Figure 1:**
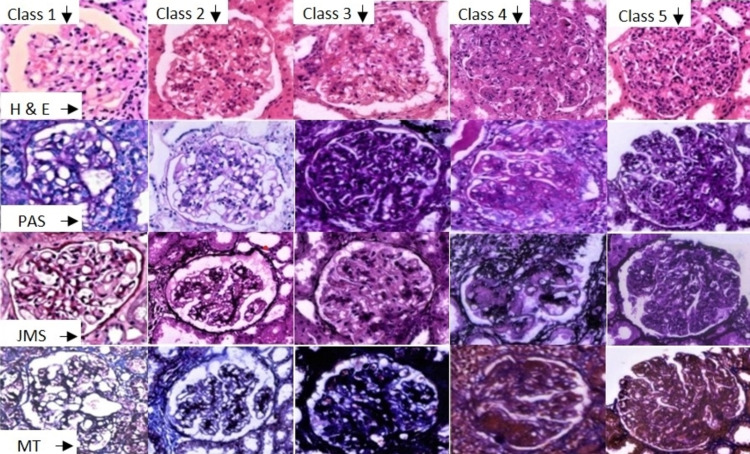
A collage of various classes of lupus nephritis Hematoxylin and Eosin (H&E) and special histochemical stains namely, Periodic Acid Schiff (PAS), Jones Methenamine Silver (JMS), Masson Trichrome (MT) is shown here. Class I is minimal mesangial LN showing normal glomerulus with no or minimal mesangial expansion. Class II is mesangial proliferative LN showing mesangial hypercellularity appreciated in H&E and JMS. Class III is focal LN showing segmental endocapillary proliferation and was noted in <50% of all glomeruli. Class IV is diffuse LN showing endocapillary and mesangial hypercellularity, cellular crescent in PAS, subendothelial hyaline deposits in JMS and fibrinoid necrosis in MT. Combined classes IV and V showed a membranoproliferative pattern of LN (×400).

**Figure 2:**
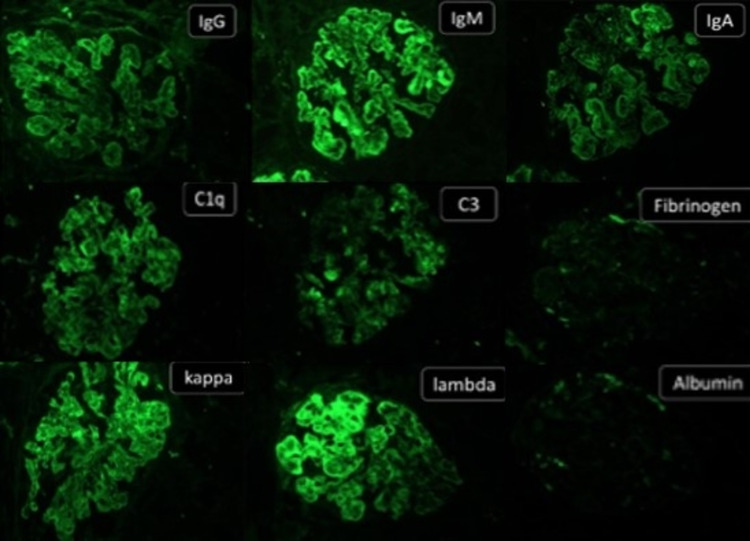
Direct immunofluorescence from a class IV LN showing full house positivity Diffuse granular capillary wall and mesangial 3+ to 4+ positivity for immunoglobulins (IgG, IgM, IgA), complements (C1q and C3), kappa and lambda light chains noted. Fibrinogen and albumin are negative (×100).

Endocapillary hypercellularity, neutrophil infiltration, fibrinoid necrosis, hyaline deposits, and interstitial inflammation were seen in a significantly higher proportion among those children having lupus nephritis with active disease than those without active disease. The chronicity index score was significant for tubular atrophy and interstitial fibrosis. The P-value was not significant in two patients with crescents and six patients with global sclerosis (Table 3). Sample light microscopy and immunofluorescence images are given in Figures 1-2, respectively.

**Figure 3:**
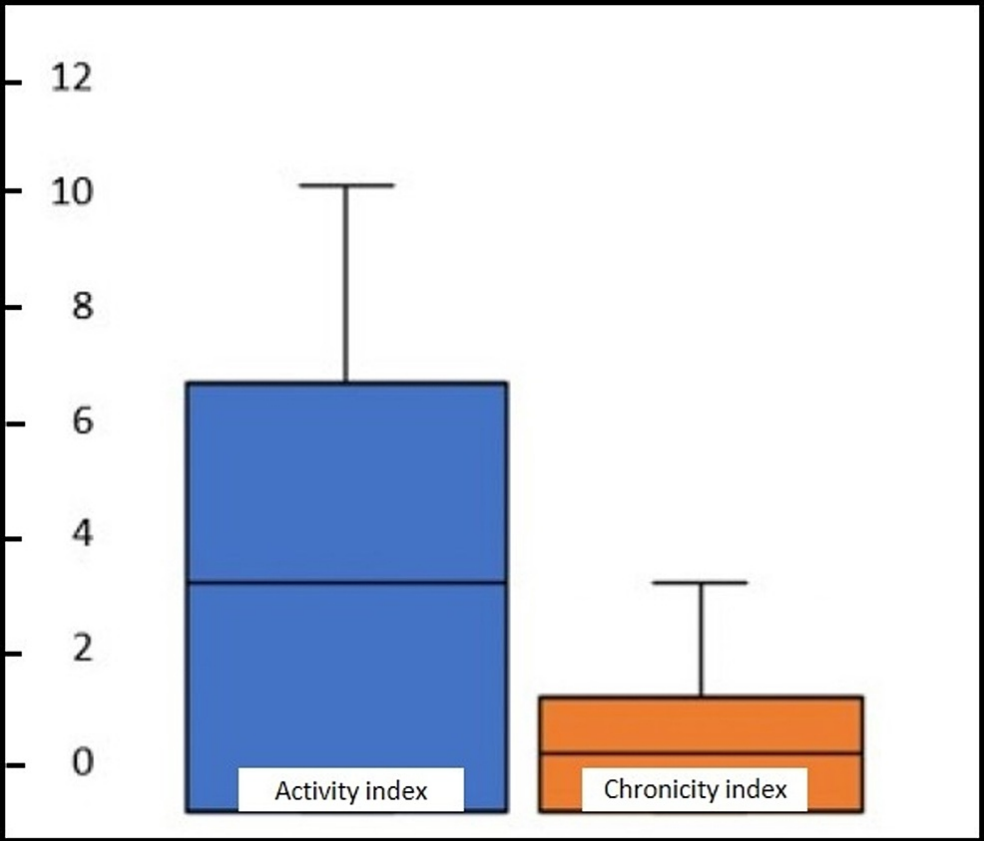
Box and whisker plot showing interquartile range of activity and chronicity score

Using box and whisker plots, the interquartile range of the activity index for classes III and IV was between 4 and 9, with the score range from 0 to 11, and the chronicity index was 0 to 4 (Figure 3).

At six-month follow-up, 35 children were under remission, 3 (7.9%) had nephritic range proteinuria and 3 (7.9%) had elevated serum creatinine with CKD stage 3. At the end of 12 months, four (10.5%) had nephritic range proteinuria, three (7.9%) had elevated serum creatinine, two with CKD stage 3, and one with CKD stage 4. Only one boy (2.6%) with class IV nephritis and CKD stage 4 succumbed due to pulmonary embolism. All children who had progressed to CKD had class 4 lupus nephritis with high activity scores ranging from 7 to 9 and chronicity scores ranging from 3 to 4. Despite treatment, renal disease progression was noted in them in the form of elevated creatinine, the persistence of proteinuria, and active urinary sediments. Though all the children had nephrotic range proteinuria during the time of renal biopsy, at the 12-month follow-up, only three children had persistent nephrotic range proteinuria.

## Discussion

Systemic lupus erythematosus is a chronic disease presenting with diverse symptoms. Genetic components are more common in childhood-onset SLE than in adult SLE. Approximately 20% of SLE cases are diagnosed before the age of 18 years [10-12]. The kidney being one of the most common organs involved, the clinical features along with the lab parameters help the treating physician to prognosticate the patient. In our study, among the 110 SLE patients who were under pediatric rheumatology care, 38 had biopsy-proven lupus nephritis. Rakesh et al. conducted a study in eastern India which showed 3.9% of children who presented to the rheumatology outpatient clinic had SLE [6]. Similar to other studies, adolescents and female children were predominantly affected, and a strong female preponderance (n=30; 78.9%) was noted overall, with the male to female ratio of 2:7.5 [13-15]. Male lupus was high in this study compared to other studies from India [3,15]. Among the renal manifestations, proteinuria was the most common (n=38, 100%), followed by anasarca (n=12; 31.5%), hypertension (n=10; 26.3%) and others. In the recent EULAR/ACR (European League Against Rheumatism/American College of Rheumatology) 2019 revised classification criteria for the diagnosis of SLE, renal biopsy evidence of lupus nephritis has been included as diagnostic criteria and a maximum weightage score of 8 and 10 has been awarded to classes II/V and III/IV LN, respectively [7]. The inclusion of renal biopsy evidence of tissue injury as a criterion for diagnosis emphasizes the importance of biopsy interpretation, classification, diagnosis, and management of LN [16,17]. In our cohort, approximately 34% of children needed a renal biopsy to stage the disease, and class IV LN was the commonest lesion (n=14, 36.8%). It was similar to a study by Mackie et al. in which 33% underwent renal biopsy and 8 out of 11 patients (72%) had class IV LN [14,15]. In the present study, the interquartile range of the activity index for classes III and IV was higher than the chronicity score. Although the maximum scores for activity and chronicity indices are 24 and 12, respectively, our study showed the scores as 0 to 11 and 0 to 4, respectively. Basu et al. compared the pediatric SLE LN with its adult counterpart and reported an activity score of 4 to 6 in class IV lupus nephritis in children and concluded that the disease was progressive in children with proliferative LN [17,18]. The activity score guides the decision to initiate aggressive immunosuppressive therapy as the outcome would be good with early initiation of therapy. They help us to know about the progression of the disease. In our study, children with classes III and IV were treated with pulse methylprednisolone 500 mg/m^2^ BSA (body surface area), followed by cyclophosphamide pulses of 500 mg/m^2^ BSA every month for six months as per the modified NIH regime. As this group is always at the risk of progression to CKD, they should be treated with aggressive immunosuppressive therapy [19]. Other ancillary treatments in our cohort included hydroxychloroquine, angiotensin-converting enzyme inhibitor therapy, and calcium supplements [20]. Depending on the extrarenal manifestations of SLE, classes I and II were treated with oral steroids at 60 mg/m2 BSA and azathioprine (2 mg/kg) or mycophenolate mofetil 600 mg/m2 BSA per dose twice a day. They were in remission at the 6th and 12th-month follow-up. Whereas in the active disease group, 4 (10.5%) had sub nephrotic range proteinuria and 3 (7.33%) had elevated serum creatinine, 2 with CKD stage 3, and 1 with CKD stage 4 at 12 month follow up. All of them had class IV LN. In a resource-restricted country like India [21], parameters like urine routine, spot urine PCR, and serum creatinine can be used instead of repeat renal biopsy as a good monitoring tool. Various studies from India described 5- to 30-year long-term survival outcomes, but our study mainly focused on the short-term treatment response [22]. On the other hand, a few studies stated that remission of proteinuria after three to six months of therapy was associated with increased long-term renal survival [23]. Overall, attaining remission was less, and the risk of progression of CKD was higher in class IV, and mortality was reported in 1 out of 38 (2.6%) in the present study. In a cohort of 54 children studied by Hari et al., 48% had class IV LN and 6% progressed to CKD 5 over 10 years [4].

The limitations of our study were the small sample size and retrospective design. The possibility of a multi-center collaboration with data on long-term follow-up may help gain a broader perspective of understanding towards childhood LN prognosis.

## Conclusions

Our study is the first on pediatric lupus nephritis from south India. We have summarized the clinical features, histopathological data of renal biopsies, and short-term outcomes of these patients. The majority of our patients developed nephritis during the course of the disease rather than at presentation and had class IV changes. At one-year follow-up, 28/38 (74%) had sustained remission. Light microscopy and immunofluorescence studies play an important role in defining the extent of renal damage in the form of activity and chronicity indices, which are the key factors in the decision-making of lupus nephritis treatment. The prognostic relevance of the histological scoring has been evaluated, and it is evident that the activity index and chronicity index go a long way in therapeutic intervention.
